# Loss of the Histidine Kinase DhkD Results in Mobile Mounds during Development of *Dictyostelium discoideum*

**DOI:** 10.1371/journal.pone.0075618

**Published:** 2013-09-25

**Authors:** Charles K. Singleton, Yanhua Xiong

**Affiliations:** Department of Biological Sciences, Vanderbilt University, Nashville, Tennessee, United States of America; Cardiff University, United Kingdom

## Abstract

**Background:**

Histidine kinases are receptors for sensing cellular and environmental signals, and in response to the appropriate cue they initiate phosphorelays that regulate the activity of response regulators. The *Dictyostelium discoideum* genome encodes 15 histidine kinases that function to regulate several processes during the multicellular developmental program, including the slug to culmination transition, osmoregulation, and spore differentiation. While there are many histidine kinases, there is only a single response regulator, RegA. Not surprisingly given the ubiquitous involvement of cAMP in numerous processes of development in *Dictyostelium* , RegA is a cAMP phosphodiesterase that is activated upon receiving phosphates through a phosphorelay. Hence, all of the histidine kinases characterized to date regulate developmental processes through modulating cAMP production. Here we investigate the function of the histidine kinase DhkD.

**Principal Findings:**

The *dhkD* gene was disrupted, and the resulting cells when developed gave a novel phenotype. Upon aggregation, which occurred without streaming, the mounds were motile, a phenotype termed the pollywog stage. The pollywog phenotype was dependent on a functional RegA. After a period of random migration, the pollywogs attempted to form fingers but mostly generated aberrant structures with no tips. While prestalk and prespore cell differentiation occurred with normal timing, proper patterning did not occur. In contrast, wild type mounds are not motile, and the cAMP chemotactic movement of cells within the mound facilitates proper prestalk and prespore patterning, tip formation, and the vertical elongation of the mound into a finger.

**Conclusions:**

We postulate that DhkD functions to ensure the proper cAMP distribution within mounds that in turn results in patterning, tip formation and the transition of mounds to fingers. In the absence of DhkD, aberrant cell movements in response to an altered cAMP distribution result in mound migration, a lack of proper patterning, and an inability to generate normal finger morphology.

## Introduction

Histidine kinases are receptors in the major mechanism of signal transduction in bacteria, the so-called two-component signaling systems that mediate numerous physiological responses to various environmental signals and conditions [1]. Most often histidine kinases are integral membrane proteins whose extracellular domain serves to recognize and bind a signaling ligand that in turn activates the intracellular kinase domain. Autophosphorylation of a histidine residue begins a phosphorelay in which the phosphate is passed to an aspartate in a downstream component, or several his-asp passes occur among multiple component proteins. The terminal aspartate acceptor is termed a response regulator whose phosphorylation activates the regulator domain, which in many instances in bacteria is a transcription factor. Various permutations of this basic signaling system exist, including histidine kinases acting as phosphatases to reverse the flow of phosphates within the relay.

Phosphorelay signaling systems have been found in several eukaryotes, including plants, yeast and fungi, and Amoebozoa [1]. *Dictyostelium discoideum* stands out among these eukaryotes because of the relatively large number of histidine kinases in its genome [2]. Several of the 15 histidine kinases of *Dictyostelium* have been characterized, and they function in a number of different processes during the multicellular developmental program, including spore encapsulation, spore dormancy, osmoregulation, prespore to spore differentiation, and the slug to culmination transition [3-11].

While the functions of and the signals/ligands that activate the histidine kinases of *Dictyostelium* are varied, all of the kinases appear to regulate phosphorelays that terminate in a single response regulator, RegA [12-14]. The regulatory domain of RegA is a cAMP phosphodiesterase, and phosphorylation of RegA by a phosphorelay activates this activity. As has been amply documented, cAMP is pervasive in mediating and regulating numerous processes during the development of *Dictyostelium* cells into a multicellular organism [15]. For instance, chemotaxis to cAMP mediates aggregation of starving individual cells resulting in the initial multicellular structure, that being a mound. Within the mounds, differential cAMP chemotaxis, along with differential cell adhesion [16], of the newly formed prestalk and prespore cell types (arising in part due to cAMP stimulation of protein kinase A) results in complex movements of the cells that generate the formation of a tip composed of various prestalk cell types [17]. The tip directs a vertical elongation of the mound into a finger and slug. cAMP chemotaxis also drives cell movements within the slugs that lead to slugs being motile [17].

Herein we characterize the function of the histidine kinase DhkD by disrupting the *dhkD* gene and observing the resulting phenotype. DhkD is a relatively large protein that possesses two histidine kinase and two receiver domains on the intracellular side of a single pass transmembrane domain and extracellular PAS and PAC domains that likely are involved in ligand binding (http://www.uniprot.org/uniprot/Q54SP4). Development of the *dhkD* null cells reveals a novel phenotype in that motility is conferred to the usually non-motile mounds. For a 3 to 4 hour period, the mounds, termed pollywogs due to their similar appearance to tadpoles, migrate randomly. Prestalk and prespore cell types are generated with normal timing and are initially scattered throughout the pollywogs as they are in wild type non-motile mounds. However, the typical patterning that subsequently results as wild type mounds form tips and the tipped mounds transition to fingers does not occur in the *dhkD* developing entities. The net result is very few fruits are formed by the mutant cells. The pollywog phenotype is rescued by disrupting the *regA* gene in the *dhkD* null cells, suggesting that *dhkD* functions by modulating the cAMP phosphodiesterase activity of RegA, like other characterized histidine kinases of *Dictyostelium* .

We postulate that in response to an as yet unknown signal, DhkD activates RegA to regulate the production of cAMP and ensure the necessary cAMP distribution within a mound. The resulting chemotactic cell movements in response to the cAMP distribution normally mediate proper patterning of the prestalk and prespore cells, tip formation, and the vertical elongation of the mound into a finger. In the absence of DhkD, we suggest that the cAMP environment within a mound is altered such that the chemotactic cell movements result in mound migration instead of finger formation.

## Materials and Methods

### Disruption of *dhkD*

A disruption construct, pdhkD-4, was made as follows. The blasticidin resistance gene cassette from pBSR519 [18] was inserted into a BamHI site between a 658 bp 5’ region of the *dhkD* gene (covering most of exons one and two) and an 812 bp 3’ region (corresponding to codons 310-580) that had been cloned into the pGEM T-easy vector (Promega). Thus, the bsr cassette replaced the first half of the first catalytic domain. The 5’ fragment was generated by PCR using primers dhkd-5 and dhkd-6, while the 3’ fragment was made with dhkd-3 and dhkd-4 (table 1). Digestion with EcoRI was carried out to release the dhkD/bsr fusion prior to transformation into *Dictyostelium* Ax4 cells via electroporation. To check for disruption, genomic DNA was isolated from blasticidin resistant clones and used as a template in a PCR reaction with a blasticidin specific primer and a *dhkD* primer downstream of the cloned 3’ region. Several disrupted isolates independently derived from multiple transformations gave the same aberrant phenotypes and showed no detectable *dhkD mRNA*. One such strain was named BS170 and was used for the experiments in this paper. Similar disruptions were also made in a strain null for *regA* (DBS0236257, *Dictyostelium* Stock Center) resulting in a strain, BS171, that is doubly disrupted in *regA* and *dhkD*. The *dhkD* disruption plasmid used in this instance was pdhkD-12 for which the bsr cassette of pdhkD-4 was replaced with a hygromycin cassette [19].

**Table 1:** Oligonucleotides used in this study.

dhkD-3	GGATCCTCAATCATTACACCA
dhkD-4	CTCTGGATTATCTTCAACCCAC
dhkD-5	TGATGGGGATACAGGAGCA
dhkD-6	GGATCCTGTTGGTCCAACAAT
dhkD-17	AGATCTCGTAGTTGTTGATATATCTTGCAT
dhkD-23	TCTAGACATTTTCACATAACACCATTTG
ACA3	TTGCTAAATCTGCCAATCCACC
ACA5	AATGGCATCTAGCTCACCATG
dcsA3	ATTTTCTCTTCCATCTCTGC
dcsA5	GGTGATTTCCCAATAAACAC
cadA5	CACTGGTGAATCATTTGAATAC
cadA3	ATTTCATATGAACCAGCAGTTG
H7Q1	ATTAGGTGGTGCCAATC
H7Q2	GTGGGCTCTTAATTGAAC

### Fusing the *dhkD* promoter to lacZ

PCR using primers dhkd-17 and dhkd-23 (table 1) gave a 1174 bp fragment corresponding to the 5’ upstream sequences and the first seven codons of the *dhkD* gene. After sequencing, the fragment was used to replace the *ecmAO* promoter in pecmAO-i-α-gal (BglII and XbaI digestion) and thus became fused to a rapid turnover version of β-galactosidase. The resulting plasmid was named pdhkD-11 and was transformed into Ax4. LacZ constructs for the pre-stalk- and pre-spore-specific promoters *ecmAO*, *ecmA*, *ecmB*, and *pspA* were generously provided by K. Jermyn and J. Williams and were transformed into Ax4 and into BS170. Staining for β-galactosidase activity was carried out as described [20].

### Cell growth and development

Strains were maintained and grown in HL-5 medium [21] at 21°C. For development, cells were grown in the presence of *Klebsiella pneumoniae* on plates, harvested, and excess bacteria removed by differential centrifugation prior to plating cells on nitrocellulose filters for standard development [22]. Plating cells at lower than normal densities or to examine slugs was done on 2% agar. For slugs, the plates were kept in a light proof box or in a box with a small pinhole to allow low intensity, directional light.

### RT-PCR

RNA was isolated using Trizol (InVitrogen). RT-PCR was carried out as described [23] using various primer pairs as listed in the results section and shown in table 1. For each primer pair, RNA concentrations, annealing temperatures, and cycle numbers were optimized to maximize sensitivity to variations in RNA levels between samples: *acaA*, 15 cycles with an annealing temperature (30 seconds) from 59° to 50° followed by 10 cycles at 55°; *cadA*, 20 cycles with an annealing temperature (30 seconds) from 55° to 45° followed by 13 cycles at 43°; *dcsA*, 15 cycles with an annealing temperature (30 seconds) from 55° to 50° followed by 10 cycles at 52°; *dhkD*, 25 cycles with an annealing temperature (45 seconds) of 55°. In each case, differences in mRNA levels of two to ten-fold were readily detected. Controls with no reverse transcriptase were included to demonstrate no genomic DNA contamination existed. Oligonucleotides specific for the H7 gene were used as an internal control as a constitutively transcribed gene during growth and development [24].

### Microscopy and image processing

Developing cells and β-galactosidase results were photographed with a Leica MZ16 stereomicroscope equipped with a Q-Imaging Retiga 1300 camera and Q-Imaging software. Images were imported into Microsoft PowerPoint, labeled, and saved as a PDF file. The figures were cropped and sized using Adobe Photoshop CS6. Time-lapse photography was carried out using a Leica DM6000B microscope with a 5X objective and SimplePCI software. 3 to 5 percent of the developing cells were expressing GFP (transformed with pTX-GFP [25]) to enhance the ability to observe cell movement. The time-lapse sequence was begun once the developing cells showed signs of obvious mound formation and was continued past the finger stage. Pictures under fluorescent light were taken at 3-minute intervals. The movies in the supporting materials show a 2.5-hour portion of the time-lapse sequence, beginning at the mound stage. The movies were compiled using 3 frames (9 minutes real time) per second.

## Results

### *dhkD* expression

**Figure 1:**
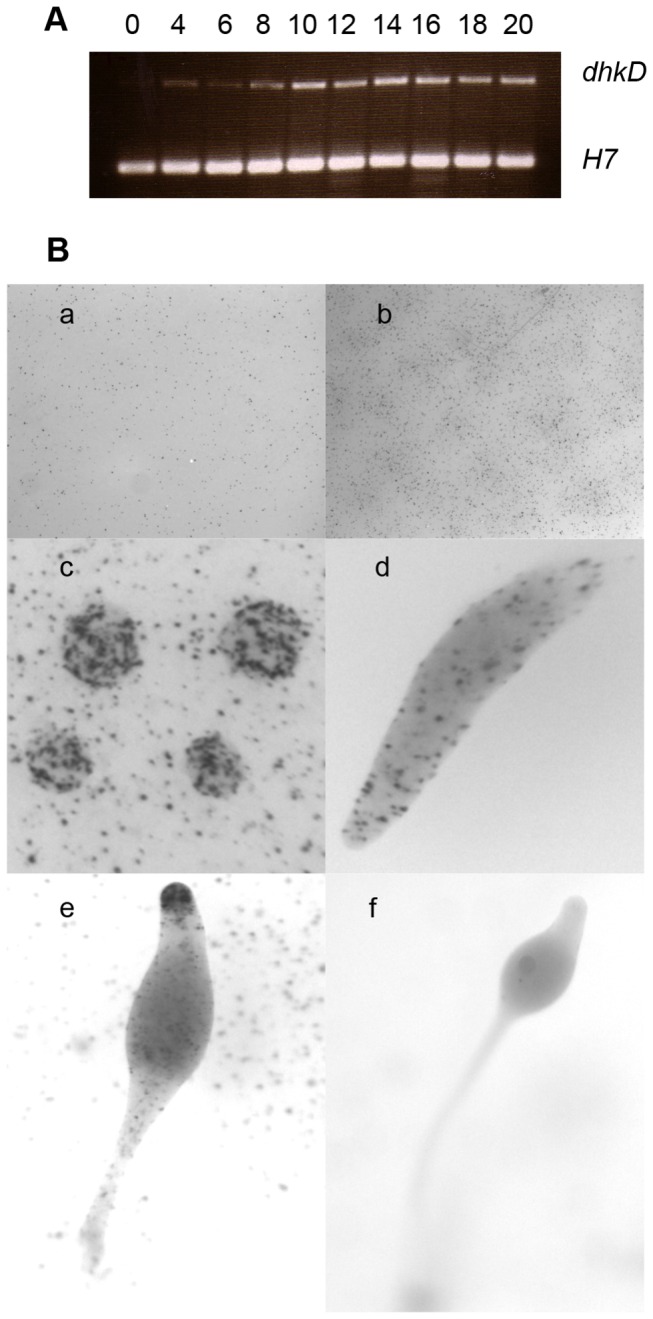
Temporal and spatial expression of the *dhkD* gene. A. RNA was isolated from Ax4 growing cells (0) and cells plated for development for the indicated times (in hours) and used in RT-PCR reactions with primers specific for the *dhkD* gene. H7 specific primers were included as an internal control as H7 is expressed constitutively during growth and development. Conditions were optimized to reveal differences in RNA levels of two to ten-fold. B. Ax4 cells transformed with pdhkD-11, which contains a fusion of the *dhkD* promoter to lacZ, were grown in the presence of bacteria, harvested, and plated for development. At appropriate times, filters of the developing cells were fixed and stained for β-galactosidase activity. All samples were stained for 18 hours at room temperature, washed, and photographed in glycerol. a, 0 hours post-starvation; b, 8 hours post-starvation; c, mound stage (12 hours); d, finger/slug stage (15 hours); e, mid-culminant stage (20 hours); f, tipped fruit (24 hours).

The histidine kinase DhkD is one of fifteen histidine kinases in *Dictyostelium* [2]. To examine the expression of the *dhkD* gene during growth and development, RT-PCR was carried out using *dhkD* specific oligonucleotides (Figure 1A). Low levels of *dhkD* mRNA were found in growing cells, with levels increasing during the first 10 hours of development as the cells aggregated and formed loose mounds. This higher level was maintained at least out to 20 or the mid culminant stage. RNA-seq data give a similar pattern of mRNA expression (http://dictyexpress.biolab.si/).

The intergenic sequence upstream of the *dkhD* gene along with the first seven codons of the coding region were fused to coding region of a rapidly degrading version of β-galactosidase, and the resulting construct was transformed into Ax4 cells in order to examine the spatial expression of the *dhkD* gene (Figure 1B). Panel a shows growing cells that have just been plated. About 5% of the cells showed detectable levels of *dhkD* mRNA. By 8 hours post-starvation (panel b), more cells were expressing *dhkD* and at higher levels. At the mound, finger, and slug stages (panels c, d), levels of expression remained about the same as at 8 hours, and the cells expressing *dhkD* were randomly distributed throughout the developing entities. During culmination (panel e), cells expressing *dhkD* were scattered within the prespore and lower cup regions and within the stalk, and there was a distinct concentration of *dhkD* expressing cells in the tips of the culminants. In tipped and mature fruits (panel f), little expression was observed in the tips, cups, and stalk, and a low, uniform expression was seen in the prespore/spore region.

### Lack of *dhkD* results in mobile mounds

**Figure 2:**
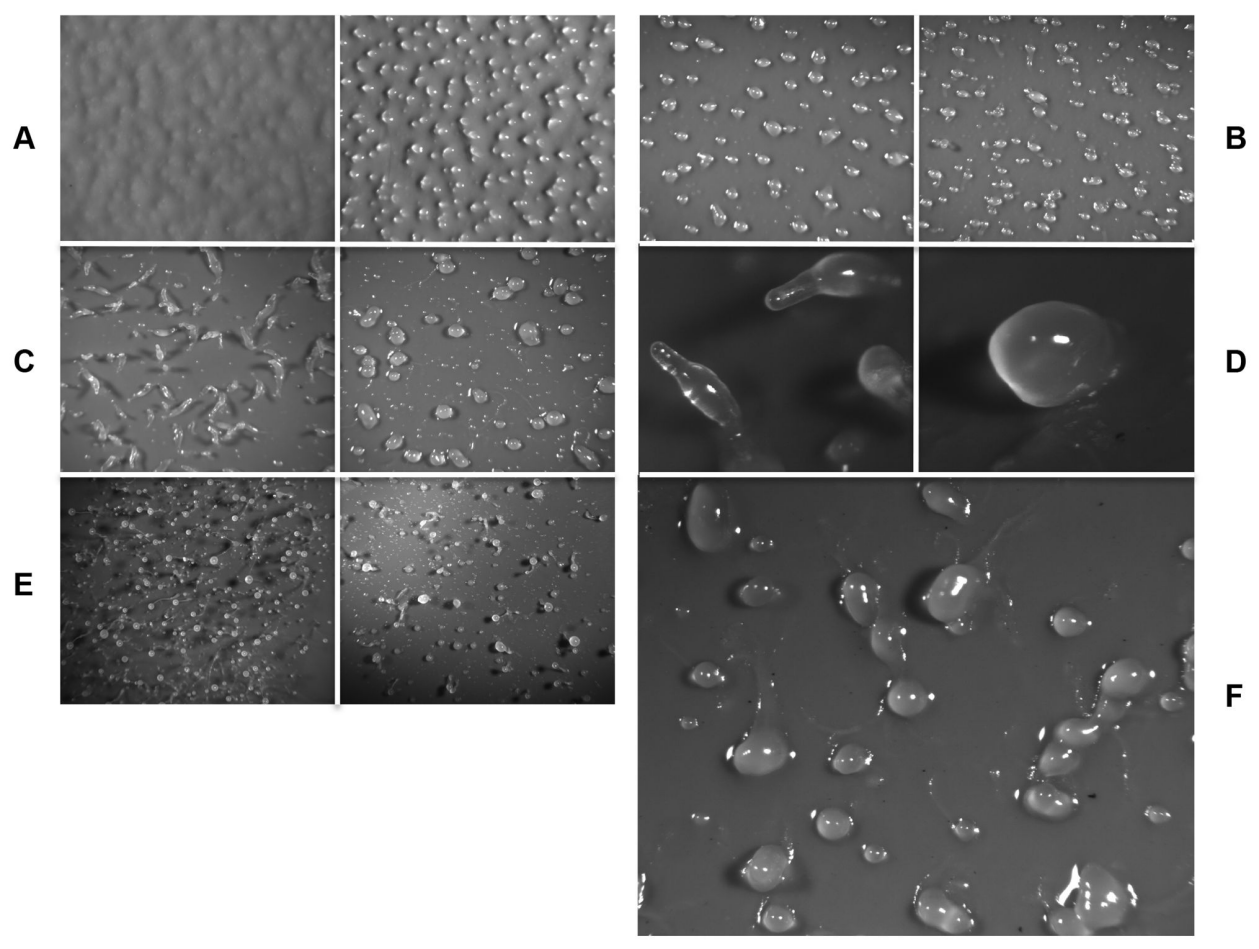
Comparison of morphology of developing Ax4 and *dhkD* null cells. Cells of each strain were grown on bacteria, harvested, and plated for development under standard conditions. For each pair of panels, the left hand picture is Ax4 while the right hand picture is *dhkD-*. A, 9 hours post-starvation; B, 12 hours post-starvation; C, 15 hours post-starvation; D, 18 hours post-starvation; E, 26 hours post-starvation; F, higher magnification of the *dhkD* null strain at 12.5 hours post-starvation.

To examine the function of DhkD, the corresponding gene was disrupted in Ax4 cells, giving rise to the *dhkD* null strain, BS170. Ax4 and BS170 cells were grown on bacteria, harvested, and plated for development. Panel A of Figure 2 shows that cells lacking DhkD form mounds 1 to 2 hours earlier than the parental Ax4 cells. Tight mounds for BS170 typically formed by 9 to 10 hours post-starvation, while mound formation for Ax4 typically occurred by 11 to 12 hours. The mounds lacking DhkD displayed a most curious phenotype. Almost immediately after forming tight mounds, the mounds became mobile and migrated for the next 3 to 4 hours. Close inspection of panel B (Figure 2) reveals trails left by the motile mounds. These are more clearly seen at higher magnification in panel F. We term the motile mounds ‘pollywogs’ based on their movement and appearance once the trails become visible. During the timeframe of the pollywog stage of the *dhkD* null cells (9-15 hours post-starvation), the parental Ax4 cells complete the formation of mounds and transition to the finger stage (Figure 2, panels B, C) by complex cell movements within a mound that results in its vertical extension.

The *dhkD*- pollywogs often collided with one another as they moved seemingly at random. About half the time, a collision resulted in the fusion of the entities, resulting in larger pollywogs. After a motile period of about 3-4 hours, the single and fused entities result in a wide range of sizes of pollywogs (panel F). Typically around 14 hours post-starvation, most, but not all of the pollywogs became stationary and appeared to attempt vertical movement or finger formation (panel C). No obvious tips formed on the pollywogs as they do on wild type mounds, and any vertical movement seemed more widespread over a large portion of the upper surfaces of the pollywogs. The structures that formed over the next few hours (15 to 18 hours post-starvation) are at best characterized as aberrant fingers with only a few looking somewhat normal. The ‘fingers’ typically were club-like or humped structures (panel D), with significant asynchrony of structures as a number of the pollywogs do not seem to change morphologically. Only rarely was there an entity that had the typical morphology of a finger or slug. In contrast, by 18 hours the parental Ax4 fingers have fully formed, undergone a transient slug stage of about an hour or so, and have initiated culmination, or the second finger stage (panel D).

Both strains underwent culmination beginning around 18 hours post-starvation. Culmination in the *dhkD* null strain resulted in a variety of morphologies, with only a small percentage resembling fruits with an obvious stalk and sorus (panel E). Even for these, the fruits were aberrant in structure, mostly with short stalks and rather large sori (perhaps due to the multiple fusions of colliding pollywogs). Other terminal structures were the humped or club-like fingers or swirled, stationary pollywogs.

**Figure 3:**
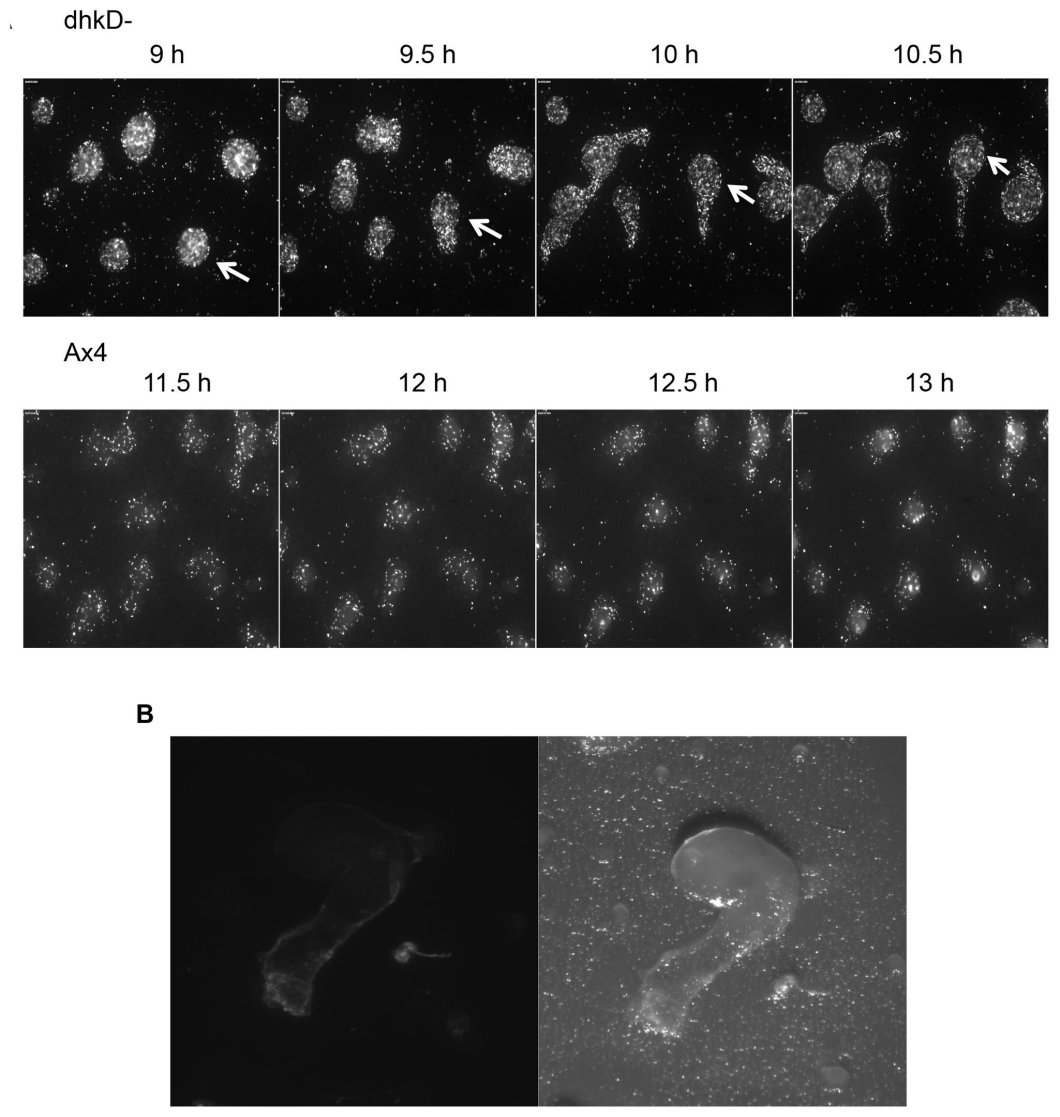
Time-lapse microscopy revels motile mounds (pollywogs) of developing *dhkD* null cells and visualization of the pollywog trails. A. Ax4 or *dhkD*- cells were grown on bacteria, harvested, and plated for development under standard conditions. 3-5 percentage points of the cells of each strain were expressing GFP. Time-lapse fluorescent microscopy was begun once the cells had formed loose mounds. Movies for the two strains can be found in the supporting materials. Four in-sequence frames, each being 30 minutes apart in real time, are shown for each strain. For the *dhkD*- frames, an arrow has been added to facilitate following the position of one of the motile mounds. B. Close-up view of a *dhkD*- pollywog (motile mound) that has been stained with calcoflour to reveal cellulose in the trail. The left panel is under fluorescent light, while the right panel is white light.

The pollywog phenotype is more clearly manifest using time-lapse microscopy. Ax4 cells or BS170 cells, with 3-5% of the cells for each expressing GFP, were plated for development and time-lapse photography was initiated as mounds began to form. Movie S1 (*dhkD-*

) and Movie S2 (Ax4) document a 2.5-hour period for each strain beginning at the mound stage. Figure 3A shows 4 in-sequence frames from each movie, with the frames being 30 minutes apart in real time. For the *dhkD* null strain, all of the mounds except the mound in the upper left corner were motile, and a clear repositioning of the mounds can been seen by comparing the 9 hour and 10.5 hour panels. As cells are left behind in the trails, the trails are easily visualized. These flattened trails, like the trails left by slugs, are composed of a slime sheath containing cellulose as revealed by calcoflour staining in Figure 3B. The panels in 3A for the Ax4 strain show no significant mound movement, and by 12.5 hours the mounds were beginning the transition to fingers by upward extension.

Examination of the movies (S1 and S2) reveals cell movement within the *dhkD*- pollywogs and Ax4 mounds. For a pollywog, the cells moved in an apparent random manner, at least at this level of analysis, during the time that the pollywog migrated. Once it became stationary, the cells within the pollywog moved rapidly (relative to movement in Ax4 mounds) in a circular manner, with either a clockwise or counterclockwise rotation. Often, the direction of rotation reversed, with more than one reversal sometimes occurring within a given pollywog. For the Ax4 mounds, the cells mostly moved circularly, again with the rotation being in either direction and with reversals. The rotation was substantially less rapid than that seen in stationary *dhkD*- pollywogs. Once the Ax4 mounds began the transition to fingers (the latter fourth of the movie), the rotational movement of the cells within the structures became more rapid and continued as such as the mounds elongated into fingers and fell over as slugs. In several of the Ax4 mounds near the end of the movie, a concentration of fluorescent cells was observed within the central upper region of the vertically elongating mounds. These are presumed prestalk cells forming the apical tip. No such concentration or tip formation was seen for the developing *dhkD*- entities.

### Slug defects in developing *dhkD* null cells

One explanation for the mobility of mounds that lack DhkD is that they have somehow precociously acquired the motility normally associated with slugs. This seems unlikely for the following reasons. First, during the development of the BS170 cells under standard conditions, slugs or slug-like entities were rarely seen. When a slug-like structure was observed, little to no migration of that entity was apparent as development progressed. In contrast, under standard conditions of development the parental Ax4 cells always showed a transient period of 1 to 2 hours of slug formation with the slugs migrating during this brief period prior to initiating culmination [6].

**Figure 4:**
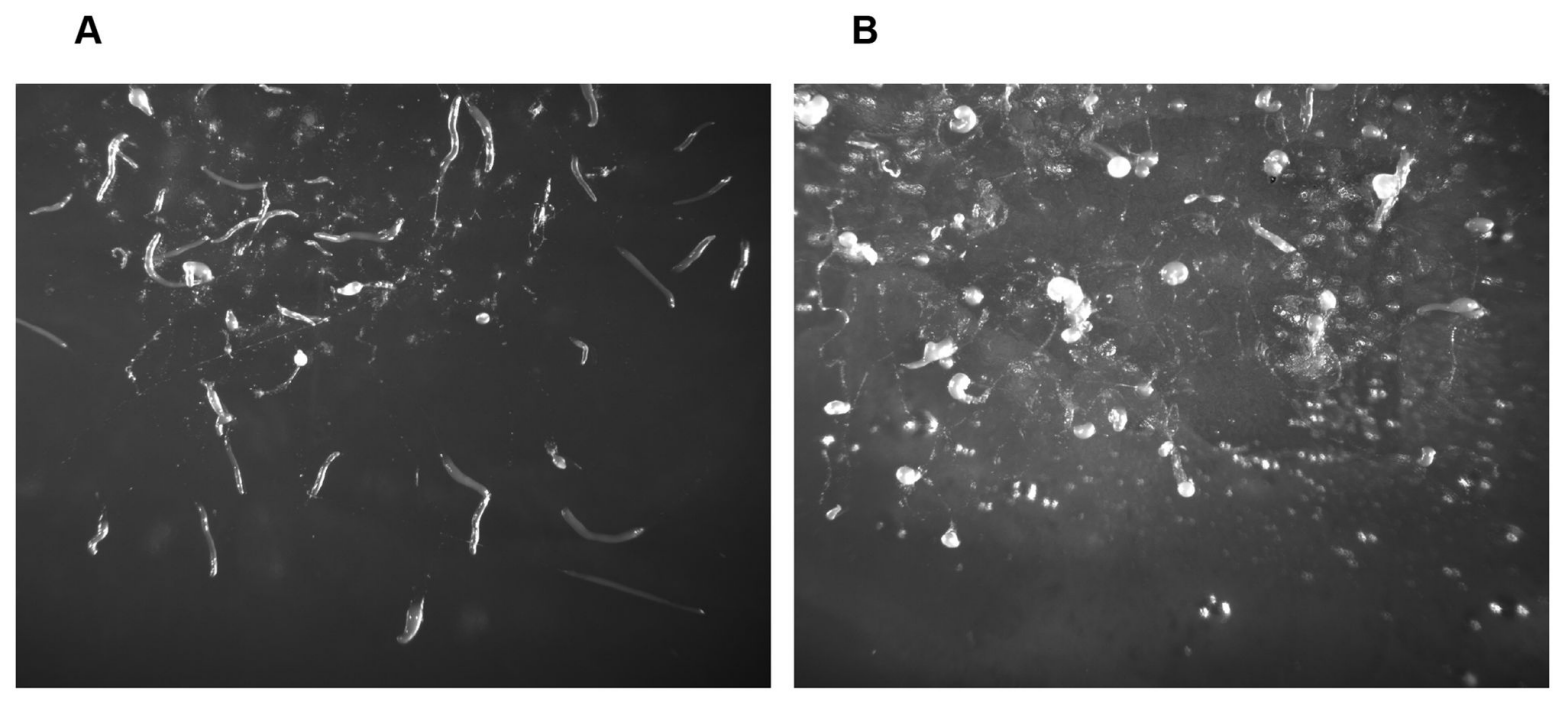
*dhkD* null cells are defective in slug formation and slug migration. Ax4 or *dhkD*- cells were grown on bacteria, harvested, and plated for development under conditions that promote slug formation (2% agar in the dark). Plates were removed after 26 hours post-starvation in the dark and were photographed. A, Ax4; B, *dhkD-*.

In addition, evidence against the pollywogs being precocious slugs was obtained when starving Ax4 or BS170 cells were plated under conditions that promote slug formation. These conditions are high humidity, no salts or buffer, and darkness. After 26 hours of starvation in the dark on 2% agar, Ax4 cells formed mostly migrating slugs as expected (Figure 4, panel A). In contrast the *dhkD* null cells formed few to no slugs. The entities observed were mostly curled pollywogs with a small number of aberrant fruits (panel B). Examination for shorter periods of time in the dark confirmed that pollywogs formed within the first 10-12 hours, followed by a period of migration the length of which was not precisely determined. Thus, the trails observed in panel B are pollywog trails. Clearly, the BS170 strain is highly defective in slug formation.

When the above experiment was repeated with a directional, weak light source instead of in complete darkness, the Ax4 slugs migrated towards the light as expected since phototaxis is a primary characteristic of slugs. Again, no slugs formed for the *dhkD*- cells, and the pollywogs showed only random migration under directional light, indicating they were not phototactic.

### *dhkD* null cells do not stream during aggregation

**Figure 5:**
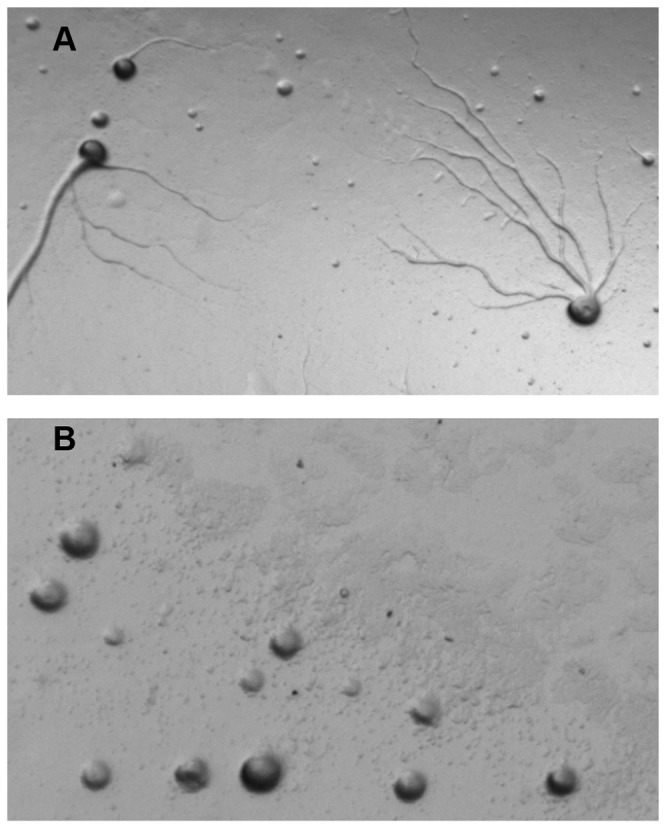
*dhkD* null cells are defective in streaming. Ax4 or *dhkD*- cells were grown on bacteria, harvested, and plated for development at ¼ the standard cells per mm^2^ on buffered 2% agar. Development was carried out with overhead light. Images are shown for each strain at 14 hours post-starvation. A, Ax4; B, *dhkD-*.

As shown above, starving BS170 cells reach the mound stage 1 to 2 hours earlier than do the parental Ax4 cells. This suggests cAMP chemotaxis in *dhkD*- cells occurs efficiently, and the precociousness of mound formation may be related to early expression of several genes involved in aggregation (described later). Nonetheless, starving BS170 cells plated at lower than normal densities revealed a defect in aggregation, or at least in the mechanism of how the cells formed mounds. Aggregation in wild type cells occurs by streaming of the cells towards cAMP being released by a forming aggregate [26]. Streams were readily observed when Ax4 cells were plated at low density on buffered agar (Figure 5, panel A), and with time a few, large mounds resulted as most cells join the first few streams that formed. In contrast, at low density streaming was not observed for starving *dhkD* null cells (panel B). The cells were able to form relatively small mounds without streaming.

### The pollywog phenotype is dependent on RegA

**Figure 6:**
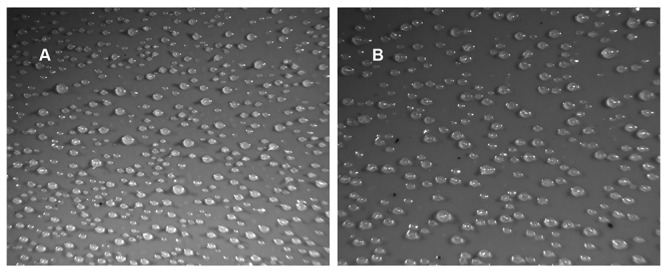
Pollywog phenotype is rescued by disruption of *regA*. The *dhkD* gene was disrupted in a *regA* null strain. The parental *regA*- and the doubly disrupted *regA-/dhkD*- strains were grown on bacteria, harvested, and plated for development under standard conditions. Images are shown for each strain at 11 hours post-starvation. A, *regA -*; B, *regA-/dhkD -*. The rapidly developing *regA* phenotype was observed for each strain with no significant morphological differences throughout development, suggesting the *dhkD*- phenotype depends on the presence of RegA.

While there are multiple histidine kinases in *Dictyostelium* , only one functional response regulator, RegA, is known. RegA is a cAMP phosphodiesterase whose activity is controlled by several different histidine kinases to regulate various aspects of development [12,13]. In order to determine if DhkD might functions through control of RegA, the *dhkD* gene was disrupted in a *regA* null strain, resulting in the doubly disrupted strain, BS171. BS171 cells were grown on bacteria, harvested, and plated for development. The characteristic ‘tipped cone’ mounds that starving *regA*- cells form also were formed by the BS171 cells, and there was no evidence of mound motility or the pollywog phenotype (Figure 6, panels A and B) as development proceeded. The precociously tipped mounds of both strains rapidly formed the typical *regA*- aberrant fingers and following rapid culmination the characteristic *regA*- aberrant fruits. Lack of mound motility was confirmed by time-lapse microscopy of the doubly disrupted strain. The results are consistent with but by no means prove that DhkD functions through the control of RegA activity.

### Wild type cells do not rescue the pollywog phenotype

**Figure 7:**
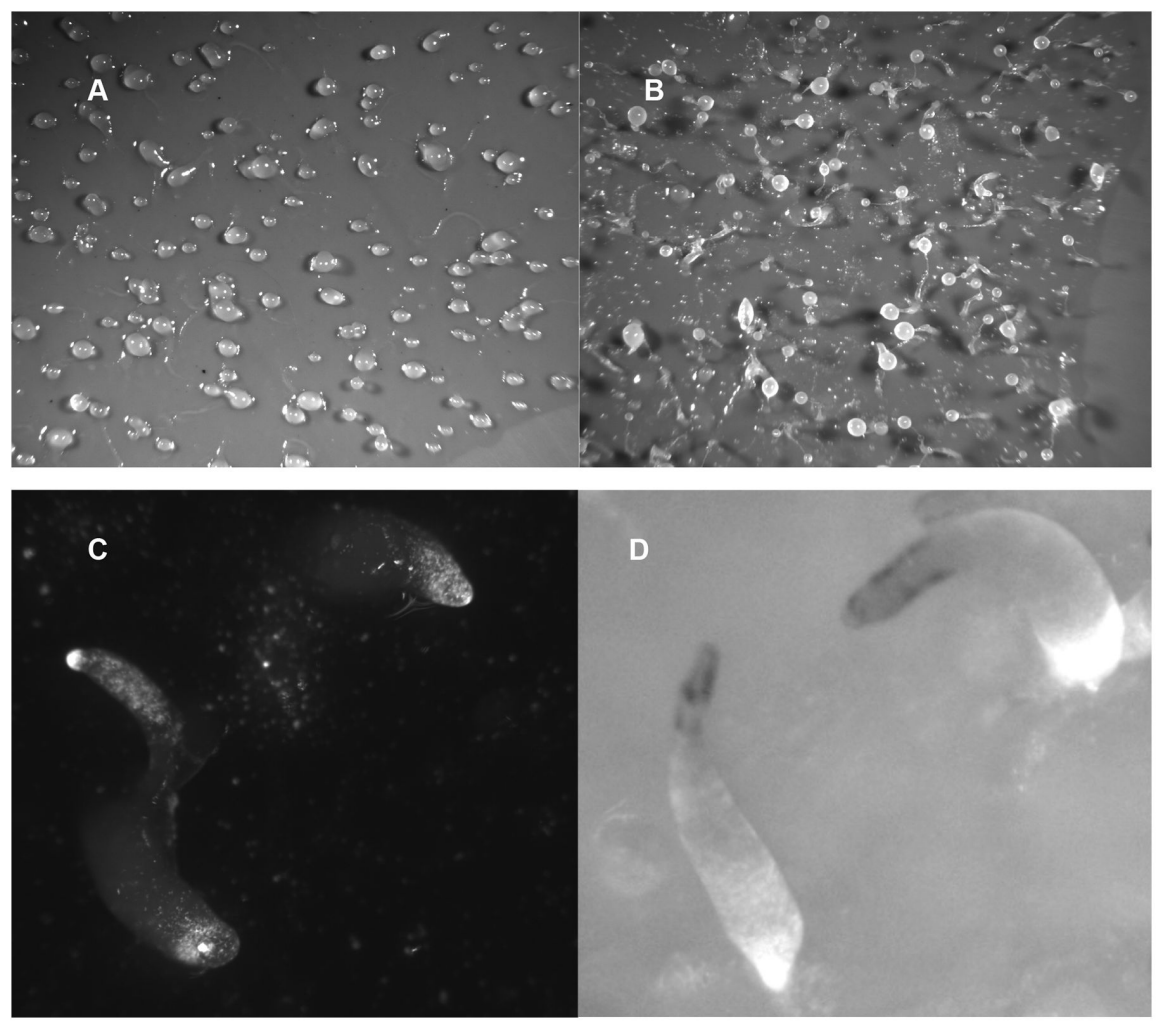
Ax4 cells do not rescue the pollywog phenotype of the *dhkD* null strain. Ax4 or *dhkD*- cells were grown on bacteria, harvested, mixed at a 1 to 1 ratio, and plated for development under standard conditions. Images are shown at 12 hours post-starvation (A) and at 26 hours post-starvation (B). In panel C, *dhkD* null cells were mixed with 3% Ax4 cells that express GFP and were plated for development. The image shown is at 15 hours post-starvation. In panel D, Ax4 cells were mixed with 5% *dhkD* null cells that express GFP and were plated for development. The image shown is at 18 hours post-starvation.

DhkD is predicted to be a membrane-bound receptor whose histidine kinase domains presumably are activated upon ligand binding. While the *dhkD* null strain likely produces the ligand, the cells should not respond as they lack the *dhkD* gene. This predicts that synergy experiments with mixes of wild type and *dhkD*- cells would not result in rescue of the *dhkD*- phenotype. This is true, at least for the pollywog phenotype. Mixing increasing amounts of Ax4 cells with *dhkD* null cells followed by starvation and plating for development did not prevent the motility of the early forming mounds. Lack of pollywog rescue was true even with mixes of 50%, as the telltale trails can be seen for the resulting mounds (Figure 7, panel A). Lack of rescue is expected if DhkD functions as a plasma membrane-bound signaling receptor as its sequence predicts. Subsequent development, however, was somewhat more normal with the percentages of fairly normal looking fingers and fruits (after culmination) increasing with increasing amounts of Ax4 cells. However, this apparent rescue of later aberrant phenotypes was not complete, even in the 50% mix of the two strains (Figure 7, panel B compared to Figure 2, panel E).

Preferential cell-type formation occurred for Ax4 cells in a background of mostly *dhkD*- cells. While initially randomly distributed within the pollywogs, the vast majority of Ax4 cells, when representing 15% or less of the total cells, appeared to differentiate into prestalk cells as reveled by their anterior or tip location in the aberrant fingers and culminants formed by the mostly *dhkD*- cells (Figure 7, panel C). When 15% or less *dhkD* null cells were mixed with a majority of Ax4 cells, the *dhkD*- cells were randomly scattered throughout mounds and early fingers. However, in mature fingers the *dhkD*- cells began to become progressively posteriorly localized and in early culminants were found only at the rear or basal disc region (panel D). As culmination progressed, the cells were lost from the structures as if they were extruded or excluded.

### Developing BS170 cells show aberrant cell type patterning

**Figure 8:**
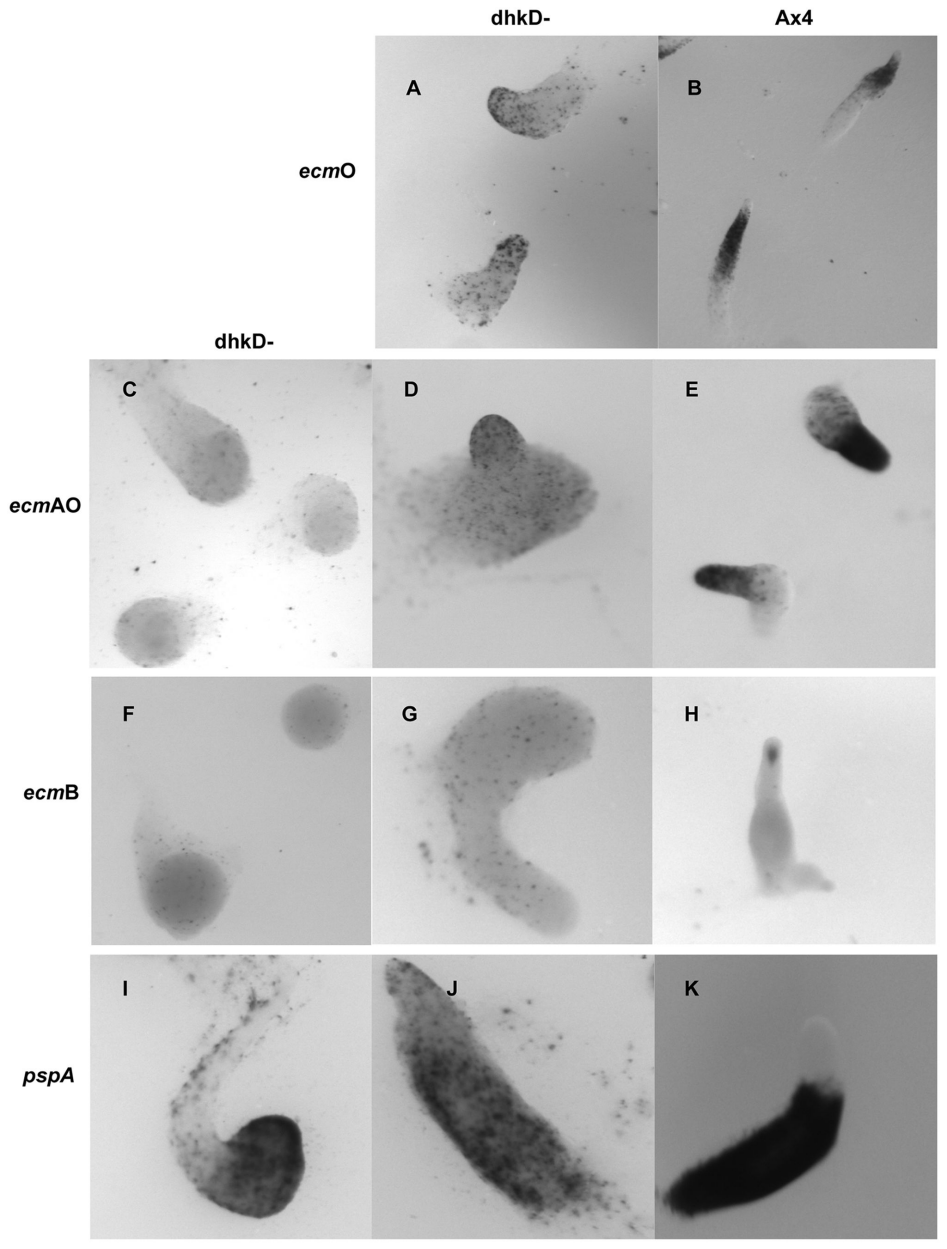
Prestalk and prespore cell formation in the *dhkD* null strain. Ax4 or *dhkD*- cells were transformed with plasmids possessing prestalk or prespore specific promoters fused to the lacZ gene. Transformed cells were grown on bacteria, harvested, and plated for development under standard conditions. At various times post-starvation, the developing structures were fixed and stained for β-galactosidase activity. For each row, the right-most panel is Ax4 and the first one or two panels are *dhkD-*. Images were taken at 38.2 X for panels A and B, and at 47.8 X for all other panels. Panels A and B, staining of prestalk O cells in *dhkD*- (A) or Ax4 (B) at the finger stage. Panels C, D, and E, staining of prestalk A and O cells in *dhkD*- (C, pollywogs; D, fingers) or Ax4 (E, fingers). Panels F, G, H, staining for prestalk B or AB cells in *dhkD*- (F, pollywogs; G, fingers) or Ax4 (H, fingers). Panels I, J, K, staining for prespore cells in *dhkD*- (I, pollywogs; J, fingers) or Ax4 (K, fingers).

Cell-type specific promoters driving expression of a labile β-galactosidase [27] were used to examine formation of prestalk and prespore cells in BS170 cells. In developing *dhkD*- cells, prestalk O cells, marked by *ecmO* expression, became apparent around 14 hours in the aberrant finger structures. However, expression was scattered throughout the structures with perhaps some concentration anteriorly (Figure 8, panel A). This contrasts with a relatively higher expression and the typical band of prestalk O cells located between the anterior tip and the posterior prespore region in Ax4 fingers (panel B).

The *emcAO* promoter was also used as it reveals both prestalk O cells and prestalk A cells, the latter occupying the anterior-most tip region of fingers. In *dhkD*- pollywogs, *ecmAO* expression was randomly scattered (Figure 8, panel C) as is typically seen in Ax4 mounds. The prestalk cells primarily become localized to the anterior tips as wild type mounds transition to fingers, as seen for the fully formed Ax4 fingers in panel E, with some anterior like cells (ALCs) scattered posteriorly. Anterior localization of prestalk A and O cells did not occur in developing *dhkD*- cells at a comparable time and stage (panel D). For the few *dhkD*- entities whose morphology more closely resembled typical Ax4 fingers, a somewhat greater degree of anterior expression of the *ecmAO* promoter was observed as development proceeded. Similar results were obtained using the *ecmA* promoter.

Prestalk B and AB cells are marked by expression of the *ecmB* promoter. Faint, scattered expression was observed early in the initial *dhkD*- mounds and in pollywogs (Figure 8, panel F), similar to the expression typically found in late, but not early, Ax4 mounds. As expected, developing Ax4 cells gave strong expression of *ecmB* in a cone of cells (prestalk AB) near the tips of fingers as culmination was initiated (panel H). Cones, and thus prestalk AB cells, were not seen at any time in the aberrant fingers and culminants of developing *dhkD*- cells (panel G). Instead, a scattered expression was maintained throughout later development, and no localized expression was seen in regions analogous to the lower cup and basal discs as is typical in Ax4 culminants and fruits.

Prespore cells are marked by *pspA* promoter expression. Expression of the *pspA* promoter was observed throughout the *dhkD*- pollywogs (Figure 8, panel I), sometimes with a concentration of expression in an outer ring of cells. In the atypical fingers, scattered expression was observed, often with some concentration in the posterior regions (panel J). The posterior bias was not overly strong, though, as it should be if prespore cells were forming and sorting normally (panel K).

### Early developmentally regulated gene expression in BS170 cells

**Figure 9:**
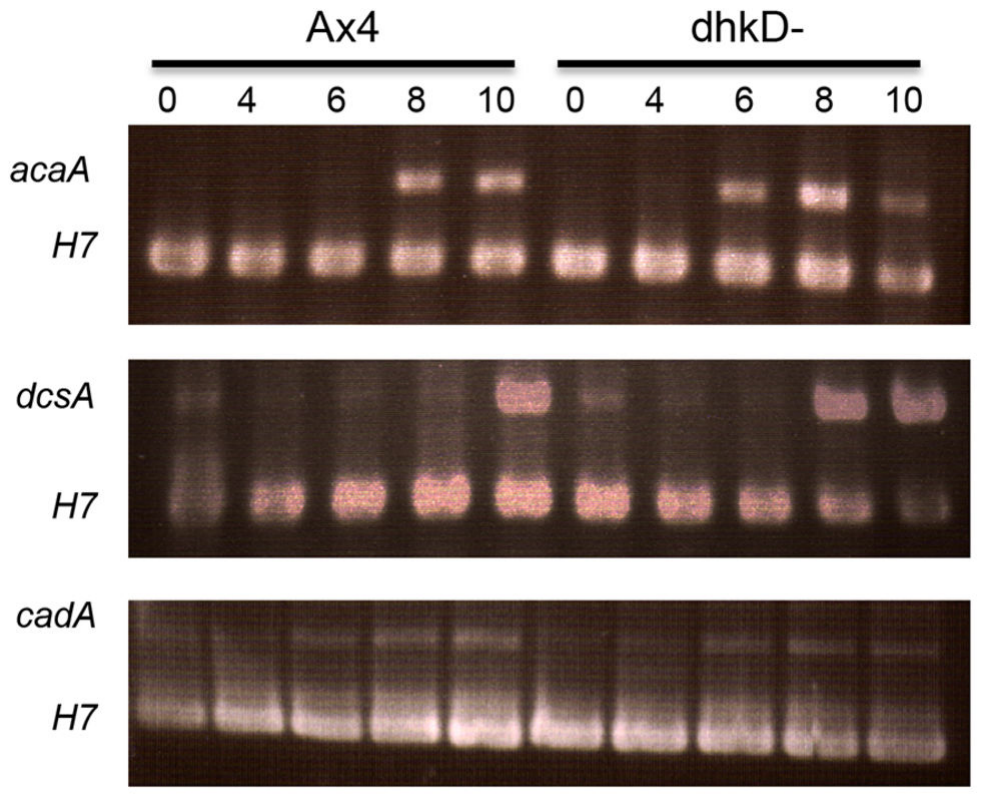
Gene expression in early developing *dhkD* null cells. RNA was isolated from Ax4 or *dhkD*- growing cells (0) and cells plated for development for the indicated times (in hours) and used in RT-PCR reactions with primers specific for various early expressed genes. H7 specific primers were included as an internal control as H7 is expressed constitutively during growth and development. Conditions were optimized to reveal differences in RNA levels of two to ten-fold. Panels are shown for the acaA gene (adenylate cyclase), the *dcsA* gene (cellulose synthase), and the *cadA* gene (gp24 adhesion protein).

Since *dhkD* null cells form mounds 1 to 2 hours earlier than Ax4 cells, the expression patterns were examined for several genes that are differentially expressed during the aggregation period. This included several genes whose protein products are involved in chemotaxis as well as genes that produce adhesion or anti-adhesion proteins. All of the examined adhesion genes (*cadA* (Figure 9), *lagC*, *csaA*, and *ampA*) were expressed similarly in developing Ax4 and *dhkD*- cells. For chemotaxis genes, *carA*, *dagA* (crac), and *gpaB* (Galpha2) were expressed normally while *acaA* was expressed two hours earlier in developing *dhkD*- cells (Figure 9). A two-hour early appearance of *dcsA* mRNA, corresponding to cellulose synthase, also was observed (Figure 9).

## Discussion

To address the function of the histidine kinase DhkD during the multicellular developmental program of *Dictyostelium* , the *dhkD* gene was disrupted. The most remarkable and intriguing phenotype associated with the loss of DhkD was the acquisition of a motile phase for mounds. Mounds formed 1 to 2 hours earlier than in the parental wild type strain, and the vast majority of the mounds immediately began migrating, leaving trails containing cellulose. Based on their mobility and appearance, these mounds were termed pollywogs. Migration of the pollywogs continued for three to four hours, after which many but not all mounds terminated their migration and transitioned over the next few hours into aberrant fingers and early culminants. Some of these were able to form more-or-less normal looking fruits, but most developing entities were unable to complete culmination and a mix of terminal structures resulted.

The motility of the pollywogs did not appear to be a precocious acquisition of the slug migration mechanism. Indeed, developing *dhkD* null cells were defective in slug formation, with only a rare slug being formed even under conditions that promote slug formation and migration [28]. Slugs that did form underwent little to no migration, and thus it seems slug migration is defective in the *dhkD* null strain. Slug migration results from complex cell movements occurring within a slug [29,30]. Cell movement within the anterior or prestalk region is best described as a scroll wave with cells spiraling around the anterior tip [31]. The scroll waves are converted into planer wave fronts in the posterior or prespore region of the slug. These complex cell movements are thought to result from chemotaxis to cAMP being produced and secreted by the anterior tip cells [32-34], with prestalk and prespore cells possessing differing chemotactic abilities [35-37]. The migration defect of *dhkD*- slugs likely reflects a disruption of the typical cell movements needed for proper slug migration.

The transition from a mound to a finger also results from complex cell movements due to cAMP chemotaxis and sorting due to differential cell adhesion [16,17,38]. Cell movement in the migrating pollywogs appeared to be random, at least at the low resolution observed herein. When a pollywog stopped migrating, the cells began a rapid circular pattern of movement, with the rotation being either clockwise or counterclockwise. The direction of rotation often reversed within the stationary pollywog. In the parental Ax4 strain, the circular pattern of cell movement was seen in loose and tight mounds, albeit at a slower rotation rate than that seen in stationary *dhkD*- pollywogs. The rotation of cells in the Ax4 mounds increased in rate as the mounds formed tips and transitioned to fingers via a vertical extension. The stationary pollywogs seemed to attempt to form fingers, but the process was not efficient and humped or club-like structures formed with only the rare observation of what could be called a tip. The time-lapse studies of Ax4 clearly showed a clustering of cells, presumably prestalk AO cells [39], and movement of the cluster to the tips of the transitioning mounds. Such clustering and tip formation was not observed in the *dhkD* null strain.

Cell movement within mounds is critical for proper sorting of prestalk and prespore cells, which initially form randomly distributed within the mound [40], and for the transition from mounds to fingers [39,41,42]. Within mounds of axenic strains, cAMP waves propagate as armed spirals resulting in a circular or rotational movement of the chemotaxing cells [41]. The greater chemotaxis of prestalk cells results in their movement to the center and top of the mound, forming the tip [17,39]. These movements lead to the generation of a twisted scroll wave of cAMP resulting in the transformation of the tipped mounds into fingers [42].

The *dhkD*- pollywog phenotype may reflect a disruption of the normal patterns of cell movement in the mounds, with the atypical movement of cells resulting in a horizontally mobile mound as opposed to tip formation and vertical extension of the mound. The histidine kinases in *Dictyostelium* that have been characterized all seem to function through the single response regulator, RegA [3,6,10,43,44]. Given that the pollywog phenotype in *dhkD* null cells was dependent upon a functional RegA, it is possible that DhkD also functions by modulating RegA activity. We postulate that DhkD modulates cAMP production in response to an as yet unidentified extracellular signal. We suggest cells expressing DhkD ensure proper cAMP waves that in turn mediate proper cell movement within mounds, and this movement typically leads to tip and finger formation. Without DhkD, distortions in cAMP production lead to altered cell movement within the pollywogs that in turn drive pollywog migration instead of tip and finger formation. It is interesting that in Ax4 mounds DhkD expressing cells did not appear to be spatially localized or patterned in a manner that might be expected for cells ensuring proper cAMP waves. Instead, DhkD positive cells were scattered throughout the mound, seemingly at random. A higher resolution and more complete study of cell movement and of cAMP wave production in pollywogs, and a more detailed examination of the movement of DhkD positive cells in wild type mounds and early fingers, are warranted.

The lack of proper patterning of prestalk and prespore cell types observed in developing *dhkD* null cells supports our proposed model of DhkD function. Timing of the appearance of each cell type in the developing *dhkD*- entities was not substantially different from that seen in the parental Ax4 cells. In contrast, though, differences in spatial patterning were observed between developing wild type and mutant cells.

Using the *ecmO* promoter as a marker, no band of prestalk O cells was observed in *dhkD*- while in Ax4 cells the typical pstO band of cells between the anterior tips and the posterior prespore region were readily apparent. Instead, *ecmO* expressing cells were scattered throughout the *dhkD*- pollywogs and in the humped finger-like structures. The *ecmAO* promoter gave a similar scattered expression pattern and thus did not reveal localized prestalk A cells that are typically found at the anterior tips of Ax4 late mounds and fingers. Lack of proper patterning also was seen for prestalk B cells, revealed by expression of the *ecmB* promoter, with only scattered cells observed in pollywogs and finger-like structures. The prestalk AB cone of cells in the tips of wild type fingers that mark the initiation of culmination were never observed in *dhkD-*. 

Together, the results with the prestalk specific promoters suggest no anterior tip or tip-like structure forms in developing *dhkD* null cells. As mentioned, a lack of tip formation was confirmed in the time-lapse studies. While prestalk cells do arise, they do not sort or localize properly. Patterning is a process dependent on differential cAMP chemotaxis and cell movements within the transitioning mounds [17,39]. Essentially the same lack of proper patterning was observed for prespore cells, although for these cells a weak posterior bias was seen. For both prestalk and prespore cells, there appeared to be fewer of each than observed in wild type developing structures, or the promoters were more weakly expressed in *dhkD*- cells. For all cell types examined except prestalk B cells, developing *dhkD*- structures whose morphology was somewhat more similar to the wild type morphology showed a more wild type patterning of prestalk cells, and some of these entities were able to form fruits, albeit usually being short and having large sori.

Examination of the expression at the level of mRNA revealed that adhesion related genes were transcribed normally in the *dhkD* null strain, namely those of *cadA*, *lagC*, *csaA*, and *ampA*. While this suggests the patterning defects do not arise from defects in cell adhesion, the production of the adhesion proteins themselves were not examined. Nonetheless, the developmental phenotype and behavior of the *dhkD* null strain was very different from that seen in mutant strains lacking or overexpressing cell adhesion proteins involved during mound and finger formation [16,45-47].

Most chemotaxis genes that were examined were expressed normally, including *carA*, *dagA* (crac), and *gpaB* (Galpha2). The exception was *acaA*, which encodes adenylate cyclase. Expression of *acaA* began about 2 hours earlier in developing *dhkD* null cells as compared to the parental Ax4 cells. Earlier than normal expression may be related to the 1 to 2 hour earlier mound formation seen in *dhkD-*. 

Normally, developing *Dictyostelium* cells stream in response to relays of cAMP pulses during the initial stages of aggregation. Near the end of aggregation, there is a change in behavior such that streaming is inefficient and instead the cells simply aggregate to complete the formation of tight mounds [48]. Interestingly, the early streaming period depends on RegA, and streams are not found in *regA* null strains [48]. It is believed that the map kinase ERK2 inhibits RegA activity during early aggregation [49], yet to date no histidine kinase has been identified that activates RegA during this time period. Given that DhkD appears to function through modulating RegA activity and that aggregating *dhkD* null cells do not stream, even at early times, it is possible that DhkD may modulate RegA activity during early aggregation and this modulation may be necessary for streaming of the cells.

## Supporting Information

Movie S1 **Developing *dhkD* null cells.** *dhkD*- cells were grown on bacteria, harvested, and plated for development under standard conditions. Time-lapse was carried out using a Leica DM6000B microscope with a 5X objective. 3 to 5 percent of the developing cells were expressing GFP to enhance the ability to observe cell movement. The time-lapse sequence was begun once the developing cells showed signs of obvious mound formation and was continued past the “finger” stage. Pictures under fluorescent light were taken at 3-minute intervals. The movie is a 2.5-hour portion of the time-lapse sequence, beginning at the mound stage. The movie was compiled using 3 frames (9 minutes real time) per second.
(AVI)

Movie S2 **Developing *Ax4* cells.** Ax4 cells were grown on bacteria, harvested, and plated for development under standard conditions. Time-lapse was carried out using a Leica DM6000B microscope with a 5X objective. 3 to 5 percent of the developing cells were expressing GFP to enhance the ability to observe cell movement. The time-lapse sequence was begun once the developing cells showed signs of obvious mound formation and was continued past the finger stage. Pictures under fluorescent light were taken at 3-minute intervals. The movie is a 2.5-hour portion of the time-lapse sequence, beginning at the mound stage. The movie was compiled using 3 frames (9 minutes real time) per second.
(AVI)
